# Genome-Wide Scan for Copy Number Variations in Chinese Merino Sheep Based on Ovine High-Density 600K SNP Arrays

**DOI:** 10.3390/ani14192897

**Published:** 2024-10-08

**Authors:** Yuezhen Tian, Jing An, Xinning Zhang, Jiang Di, Junmin He, Ayinuer Yasen, Yanpin Ma, Gaohaer Sailikehan, Xixia Huang, Kechuan Tian

**Affiliations:** 1 Key Laboratory of Genetics Breeding and Reproduction of Xinjiang Cashmere and Wool Sheep, Institute of Animal Science, Xinjiang Academy of Animal Science, Urumqi 830011, China; 2 College of Animal Science and Technology, Northwest Agriculture and Forest University, Yangling, Xianyang 712100, China; 3 College of Animal Science, Xinjiang Agricultural University, Urumqi 830052, China; 4 Institute of Animal Science and Veterinary Medicine, Shandong Academy of Agricultural Sciences, Jinan 250100, China

**Keywords:** copy number variations, fine wool sheep, Ovine HD BeadChip

## Abstract

**Simple Summary:**

A genome-wide copy number variations (CNVs) analysis using high-density Ovine BeadChip array data in 288 Chinese Merino sheep was conducted. A total of 656 CNV regions (CNVRs) with an average size of 66.88 Kbps were identified. These CNVRs contain 519 losses, 60 gains, and 77 gain–losses ranging from 1.5 Kbps to 646 Kbps and covering 43 Mbps (1.58%) of the whole Ovine genome. To validate these results, we performed a quantitative PCR to detect 11 randomly selected CNVRs and successfully verified 8 (72.7%). The gene functional enrichment analysis revealed that a total of 1592 genes in 465 CNVRs were identified as related to biological functions such as ATP binding activity. Our results expand the current CNV map of the sheep genome and provide preliminary fundamental information for carrying out CNV studies of fine wool sheep in the future.

**Abstract:**

Sheep are a vital species in the global agricultural economy, providing essential resources such as meat, milk, and wool. Merino sheep (Junken type) are a key breed of fine wool sheep in China. However, research on fine wool traits has largely overlooked the role of SNPs and their association with phenotypes. Copy number variations (CNVs) have emerged as one of the most important sources of genetic variation, influencing phenotypic traits by altering gene expression and dosage. To generate a comprehensive CNVR map of the ovine genome, we conducted genome-wide CNV detection using genotyping data from 285 fine wool sheep. This analysis revealed 656 CNVRs, including 628 on autosomes and 28 on the X chromosome, covering a total of 43.9 Mbs of the sheep genome. The proportion of CNVRs varied across chromosomes, from 0.45% on chromosome 26 to 3.72% on chromosome 10. Functional annotation through Gene Ontology (GO) and Kyoto Encyclopedia of Genes and Genomes (KEGG) pathway analyses highlighted significantly enriched GO terms, including odorant binding, ATP binding, and sulfuric ester hydrolase activity. The KEGG analysis identified involvement in pathways such as neuroactive ligand–receptor interaction, axon guidance, ECM–receptor interaction, the one-carbon pool by folate, and focal adhesion (*p* < 0.05). To validate these CNVRs, we performed quantitative real-time PCR experiments to verify copy number predictions made by PennCNV software (v1.0.5). Out of 11 selected CNVRs with predicted gain, loss, or gain–loss statuses, 8 (IDs 68, 156, 201, 284, 307, 352, 411, 601) were successfully confirmed. This study marks a significant step forward in mapping CNVs in the ovine genome and offers a valuable resource for future research on genetic variation in sheep.

## 1. Introduction

Copy number variations (CNVs) refer to structural variants where specific DNA segments have an altered number of copies, ranging from 1 kilobase (Kb) to several megabases (Mbs) [1,2]. Compared to single nucleotide polymorphisms (SNPs), CNVs can have a greater impact on phenotypic variation by altering gene expression and dosage. CNVs can be either deleted or duplicated through translocation, insertion, or deletion [2,3], making them a significant source of genetic variation between generations and phenotypic diversity among individuals and populations [4,5,6]. Numerous CNV studies have been conducted on humans [7,8,9], mice [10,11,12,13], dogs [14,15,16], cattle [17,18,19,20], chicken [21], pigs [22], goats [23], and sheep [24]. For example, in the human population, alterations in the number of copies of the *CCL3L1* gene have been found to be linked with susceptibility to HIV-1/AIDS [25,26] and *FCGR3B* copy number variation has a significant effect on systemic lupus erythematosus (SLE) [27]. Additionally, CNVs have been observed in domestic animals, leading to variations in phenotype; for instance, one copy of a 450 kb duplication in the *KIT* gene associated with white coat phenotype in pigs [28]. Furthermore, a 4.6 Kbp duplication in intron 6 of *STX17* influences the phenotype of hair greying and melanoma in horses [29]. In sheep, the gain-type CNV of *PIGY* has shown better growth traits than other types [30]. Above all, all these studies have demonstrated that CNVs are valuable genetic variant resources for understanding complex phenotypes and economically important trait variations in domestic animals.

CNV detection is primarily done using four platforms: comparative genomic hybridization (CGH) arrays, next-generation sequencing (NGS) [31], long-read sequencing [32], and SNP arrays [6]. Although CGH arrays have excellent signal-to-noise ratios [33,34], they are not suitable for large-scale CNV detection, especially on a genome-wide scale. While NGS provides a more detailed characterization of CNVs [2,3,19,35], it remains too expensive for CNV detection in large populations. On the other hand, SNP arrays offer a more desirable performance in genome-wide CNV detection and are more cost-effective, enabling users to increase the number of samples at a reasonable cost. However, the primary limitation of SNP arrays for CNV detection is the low density of SNP coverage, making it difficult to detect small CNVs [36]. This limitation can be mitigated to some extent by using high-density SNP arrays. Therefore, high-density SNPs have been widely and successfully used in detecting CNVRs in human and various animal genomes. 

Sheep (*Ovis aries*) are among the earliest domesticated livestock species, providing resources like meat, milk, and wool. These contributions have made sheep a vital part of the global agricultural economy since the Neolithic era [37,38]. Merino sheep (Junken type) is a major breed of fine wool sheep in China, bred since 1972 [33]. In the Xinjiang Uyghur Autonomous Region, where Chinese Merino sheep are raised, wool typically contributes to 21% of the total flock income [39]. They are known for their soft, high-quality wool, highly valued in the textile industry. Over the past few decades, improving wool economic traits in Merino sheep has been a major focus in many countries, utilizing various quantitative and marker-assisted methods to identify quantitative trait loci (QTL) associated with wool traits [40]. Wool keratin-associated proteins (KAPs) are structural elements of wool and hair fibres, creating a matrix that encases the keratin intermediate filaments (KIFs). They are thought to be crucial in determining the physical and mechanical properties of wool fibres [41,42]. The *KAP6* gene has been reported to be linked to Mean Fibre Diameter (MFD) in medium wool Peppin Merino populations [43,44]. A 57 bp deletion in *KRTAP6-1* was linked to the production of coarser wool, characterized by a higher Fibre Diameter Standard Deviation (FDSD), an increased Coefficient of Variation of Fibre Diameter (CVFD), and a greater Prickle Factor (percentage of fibres exceeding 30 microns; PF) [45]. It has been reported that *KRTAP1-2* influences clean fleece weight (CFW), greasy fleece weight (GFW), and yield in Merino cross lambs [46]. Variation in *KRT19-5* was linked to the mean fibre curvature of wool in Chinese Tan Sheep [47]. *KRT31* has been reported to be associated with increases in GFW, CFW, and mean staple length (MSL) [48]. The *KRT34* promoter variation may influence MFD, FDSD, and MSL [49]. *KRT81* may impact wool growth by modifying the density of wool follicles in the skin, the density of individual fibres, or the skin area producing fibres, rather than by changing the rate of fibre extrusion or their diameter [50]. *KRT84* showed high expression in hair follicles, particularly in the inner root sheath, outer root sheath, and hair medulla, and was consistently expressed across all six lamb ages studied, from 1 to 270 days [51]. However, most of these studies are based on polymorphisms and their association with wool quality traits, and the impact of genome-wide CNV landscapes on Merino sheep and their influence on phenotypic variation remains largely unknown.

The main objective of this research is to identify CNVs in Chinese merino sheep. The study analysed data from 288 animals using high-density 680K SNP genotype arrays and the PennCNV program [52]. Furthermore, the CNVRs were verified using the qPCR strategy. This is the first CNVR map of fine wool sheep constructed using high-density SNP genotype data. This study presents a detailed CNVR map of the ovine genome, laying the groundwork for future association studies linking CNVs to economically significant traits in fine wool sheep. 

## 2. Materials and Methods

### 2.1. Ethics Statement

Blood samples were collected from 288 fine wool sheep in strict accordance with the guidelines approved by the Biological Studies Animal Care and Use Committee of Xinjiang Province for animal experiments in the People’s Republic of China. All efforts were made to minimize any discomfort during blood collection. Sample collection and experimental protocols were performed in strict accordance with the guidelines approved by the Biological Studies Animal Care and Use Committee of Xinjiang Province for animal experiments in the People’s Republic of China (Approval Number XAASA-20180110).

### 2.2. Sample Collection and Genotyping

A total of 288 fine wool sheep were randomly selected from the original breeding stock of Chinese Merino sheep (Xinjiang type) in the eastern Yili River Valley, Xinyuan County (43°03′–43°41′ N, 82°28′–84°56′ E), Xinjiang Province, China. A volume of 2–3 mL of whole blood was collected per animal in EDTA anticoagulant tubes from the sheep jugular vein, aliquoted into 2 mL centrifuge tubes and frozen at −20 °C for genomic DNA extraction. Genomic DNA was extracted using a TIANamp Blood DNA Kit. The 288 samples were genotyped using the Illumina Ovine SNP680K BeadChip, which includes 606,006 SNPs across the ovine genome, with an average spacing of 4.28 Kbs. The genotyping platform used was Illumina’s Infinium II Multi-Sample Assay and the SNP chips were scanned using iScan and analysed using Illumina’s INFINIUM GENOMESTUDIO software (Version 2009.1). To increase the accuracy of CNV inference, stringent quality control criteria were applied to filter out low-quality SNPs (individual call rate > 90% and SNP call frequency > 90%). 

### 2.3. Data Quality Control and CNV Calling 

The genomic DNA of 288 individual samples from a fine wool sheep breed (Chinese merino sheep Xinjiang type) was genotyped using the Illumina high-density 680K SNP BeadChip following the manufacturer’s protocol. For each SNP marker, log R ratio (LRR) and allelic intensity (B allele frequency: BAF) ratio were exported using Illumina GenomeStudio software (v2.0.5). The population frequency of the B allele (PFB) file was then calculated based on the BAF for each marker. To identify CNVs in the fine wool sheep genome, we employed the PennCNV software [52] http://www.openbioinformatics.org/penncnv (accessed on 9 January 2023) from SNP chip data on both 26 autosomes and chromosome X. To overcome the status of ‘genomic waves’, we generated the sheep GC model file by calculating the GC content of the 1 Mb genomic region surrounding each marker (500 Kbs on each side) and then used the ‘-gcmodel’ option of PennCNV to adjust the results. PennCNV used a hidden Markov model (HMM)-based approach to model the dependence structures between copy numbers. It then optimized the HMM parameters using the Baum–Welch algorithm, and we selected the final CNV list based on the posterior probabilities. To reduce the false discovery rate in CNV calling, we adopted the following criteria: (1) the standard deviation (SD) of LRR should be less than 0.30; (2) the BAF drift should be 0.01, and the waviness factor value should be between −0.05 and 0.05; and (3) the CNV should contain ten or more consecutive SNPs. Finally, the CNV regions (CNVRs) were determined by aggregating the overlapping CNVs identified across all samples according to previously published protocols [5]. Following the requirements of PennCNV, we defined CNV call filtering criteria and excluded samples with low-quality signal intensity data. Ultimately, a total of 285 samples remained for further CNV analyses.

### 2.4. qPCR Confirmation of CNVRs

Quantitative PCR (qPCR) was performed to validate 11 randomly selected CNVRs identified by PennCNV. Following previous methodologies [53], the glyceraldehyde-3-phosphate dehydrogenase gene (GAPDH), known to be present in a single copy across various species, was chosen as the reference gene. Primers were designed using National Center for Biotechnology Information (NCBI) reference sequences via the Primer 3.0 web tool (https://bioinfo.ut.ee/primer3/), as shown in Supplementary Table S3. qPCR reactions were carried out on a BIO-RAD CFX98™ Real-Time System following the manufacturer’s instructions and recommended cycling conditions. Each sample was subjected to triplicate reactions using a 20 µL volume containing 1 µL of DNA (approximately 50 ng), 2 µL of forward and reverse primers (concentration 0.1–0.75 µM), 10 µL of 2× Master Mix, and 15 µL of water. All qPCRs were carried out in 96-well reaction plates under the following conditions: initial denaturation at 95 °C for 3 min followed by 40 cycles of denaturation at 94 °C for 10 s and combined annealing and extension at 60 °C for 30 s. The 2^−ΔΔCt^ method was employed to calculate the copy number [54], where Ct represents the threshold cycle. The corresponding equation follows the method reported by Zhu et al. [24]. Analyses of 3 technical replicates of the same DNA samples and 2 negative control samples in the same batch were performed, and then the coefficient of variation (CV%) between reaction tubes of each sample in the same group was calculated to exclude the time effect.

### 2.5. Functional Enrichment Analysis Procedures for CNVRs 

Gene information was retrieved from the BioMart data management system (http://asia.ensembl.org/biomart/martview/) based on the *Ovis aries* (Oar_v3.1) genome assembly. To gain insight into the functional implications of genes overlapping or within CNVRs, Gene Ontology (GO) annotation [55] and Kyoto Encyclopedia of Genes and Genomes (KEGG) pathway analyses [56] were conducted using the online software DAVID (http://david.abcc.ncifcrf.gov/) (v2023q4) [57]. Due to the limited annotation of the sheep genome, *Ovis aries* homologous Ensembl gene IDs were converted into human orthologue Ensembl gene IDs for analysis. BH-corrected FDR *p*-value of 0.05 was used as the criterion for determining significance levels.

## 3. Results

### 3.1. CNV Genotyping

**Figure 1:**
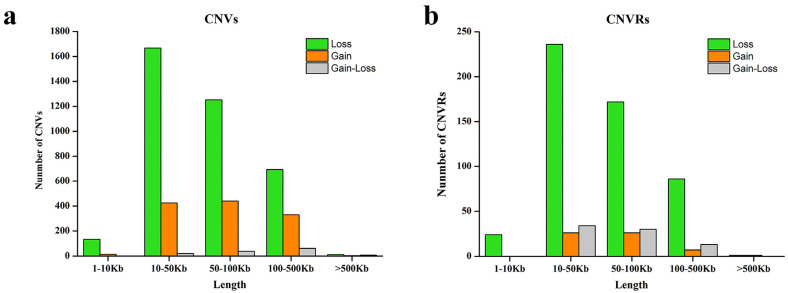
Size distribution of copy number variations: (**a**) CNV size distribution in fine wool sheep and (**b**) CNVR size distribution in fine wool sheep.

A total of 5101 CNV events were identified after excluding unreliable CNV calls in 285 Chinese fine wool sheep, consisting of 3760 losses, 1214 gains, and 127 gain–loss events (Additional file: Table S1). The CNVs had an average length of 76.1 Kbs and a median length of 55.2 Kbs (Table 1). Additionally, around 41.44% of the CNVs were between 10 and 50 Kbs in length, while 33.93% ranged from 50 to 100 Kbs, with only approximately 3% being small fragment CNVs (<10 Kbs) (Figure 1a). After consolidating overlapping CNVs, a total of 656 CNVRs were identified, including 628 autosomal and 28 X chromosome CNVRs, covering 43.9 Mbs of the sheep genome and accounting for 1.58% of the entire genome (Additional file: Table S2). These CNVRs ranged in size from 1.6 Kbs to 646.2 Kbs, with an average length of 66.9 Kbs and a median of 51.1 Kbs (Figure 1b). Within these 656 CNVRs, 519 loss events, 60 gain events, and 77 gain–loss events were observed. The number of loss events was approximately 8.65 times greater than that of gain events, consistent with findings in other species [22,53,58]. Additionally, a total of 653 CNVRs were found in at least two samples, 11 were identified in more than 50 samples, and 125 CNVRs were identified in more than 10 sheep.

### 3.2. Genome-Wide Surveys of CNVs and CNVRs in Fine Wool Sheep

**Figure 2:**
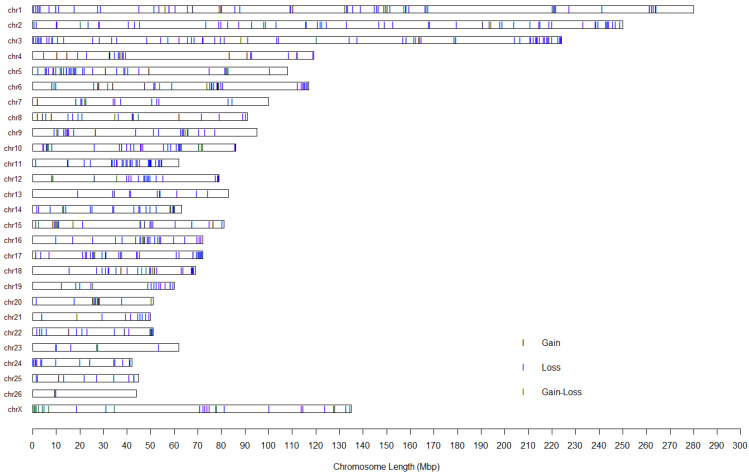
Map of CNVRs in fine wool sheep. Black indicates gains, blue represents losses, and dark green denotes regions with both gain and loss events.

We produced a map illustrating the distribution of CNVRs across the chromosomes in fine wool sheep (Figure 2). Our findings revealed a non-random distribution of CNVs across the various chromosomes. The percentage of CNVRs on the chromosomes varied, ranging from 0.45% on chromosome 26 to 3.72% on chromosome 10, with the number of CNVRs ranging from 2 on chromosome 26 to 66 on chromosome 3.

### 3.3. CNV Validation by Quantitative PCR

**Figure 3:**
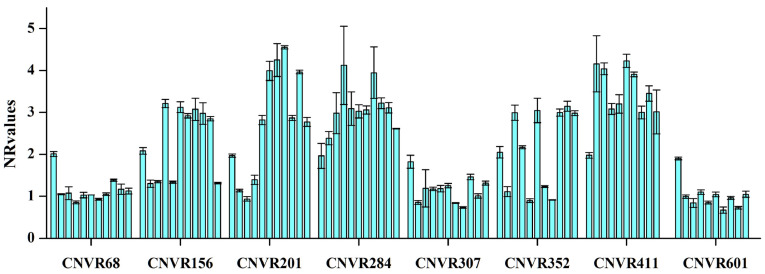
Normalized ratios (NRs) obtained by qPCR for 8 CNVRs. The *y*-axis displays NR values from qPCR, while the *x*-axis represents the different CNV regions. Samples with NRs of around 1 indicate individuals with a single copy (copy loss), NRs of around 2 represent normal individuals (two copies), and NRs of 3 or higher signify individuals with copy number gains.

We performed quantitative real-time PCR experiments to evaluate the accuracy of the copy number designations identified by PennCNV [52]. We chose 11 potential CNVRs that represented various predicted copy number statuses (gain, loss, or gain–loss) for CNV validation. Our findings indicated that eight of these CNVRs (CNVR IDs = 68, 156, 201, 284, 307, 352, 411, 601) were successfully validated (see Figure 3). Further details regarding the primers and results can be found in the Supplementary Information (Additional files: Tables S3 and S4).

### 3.4. Functional Enrichment Analysis of CNVRs

The Ensembl Genes Database from the Biomart data management system was utilized to retrieve the gene content of 656 CNVRs. A total of 1592 annotated genes were identified within 467 CNVRs in fine wool sheep. Among these genes, 1530 are protein-coding genes, 32 are snRNAs, and the remainder include pseudogenes, small nucleolar (snoRNA) genes, and rRNAs. Detailed information on these genes is provided in Additional file Table S5.

To assess the functional annotation of these CNVRs, we conducted GO and KEGG pathways analyses using the DAVID tool. A total of 31 GO terms and five KEGG pathway terms were statistically significant after Benjamin correction (Additional file: Tables S6 and S7). The significantly enriched GO terms were associated with specific biological functions such as odorant binding, ATP binding, and sulfuric ester hydrolase activity. The KEGG analysis confirmed that these genes are primarily involved in neuroactive ligand–receptor interactions, axon guidance, ECM–receptor interactions, one-carbon pool by folate, and focal adhesion (*p* < 0.05). Moreover, we identified genes associated with hair follicle development and fibre diameter traits, including Keratin, Type I Cytoskeletal 17 (*KRT17*), Mitogen-Activated Protein Kinase 1 (*MAPK1*), and EPH receptor A5 (*EPHA5*), which overlapped with CNVRs identified in this study.

## 4. Discussion

In recent years, CNVs have become increasingly recognized as a significant source of genetic variation, playing a crucial role in phenotypic diversity related to complex diseases in humans and economically important traits in livestock. Additionally, in the field of cancer, CNVs can be used for early diagnosis and detection because they are often involved in specific chromosome segments with relatively strong intensity [59]. For example, an algorithm has been developed to determine cancer types based on the CNVs, aiding in early cancer diagnosis [60]. 

With the advancement of high-density genotyping arrays, CNV detection using SNP genotyping arrays has become an efficient approach. Several algorithms for CNV inference based on SNP chip data have been developed, such as PennCNV, cnvPartition, GADA, and QuantiSNP. Each algorithm has its strengths and weaknesses [36]. Despite the potential for more reliable results by using multiple algorithms, many studies on CNV detection using SNP arrays in humans and animals have only utilized the PennCNV software (v1.0.5) [22,23,61,62,63]. In our study, we chose to use PennCNV to detect CNVR for two reasons. Firstly, the PennCNV algorithm incorporates various sets of information, including the total signal intensity of LRR, the distance between neighbouring SNPs, the BAF, and the population frequency of PFB [64]. Additionally, PennCNV employs a computational method that incorporates regression models with GC content to mitigate genomic waves, ensuring greater reliability compared to other algorithms. Secondly, using multiple algorithms to detect CNVs can make it difficult to determine the appropriate number of CNVs. Relying solely on commonly detected CNVs can lead to missing true CNVs, while accepting all CNVs identified by different software may result in numerous false positives. To minimize the risk of false positives associated with using a single algorithm, we applied two stringent criteria (SD of LRR < 0.30 and BAF drift = 0.01). These rigorous standards contributed to a high qPCR validation rate of 72.7% in our study.

In this study, we used the Ovine high density (HD) SNP and identified a total of 653 CNVRs, including 28 CNVRs on the X chromosome. The frequencies of these detected CNVRs are not high, only 11 CNVRs are present in more than 50 samples, accounting for 17.3% of the total samples. Similar results have been identified in other CNV detection studies. Yang et al. (2018) conducted a CNV detection study using ovine SNP50 Beadchip data on 2254 geographically dispersed sheep and identified a total of 619 CNVRs [65]. Wu et al. (2015) performed a CNV detection study using Illumina Bovine HD BeadChip (770k) data on 792 Simmental cattle and identified a total of 263 CNVRs, with only 14 of these CNVRs identified in more than 100 individuals (12.6%) [20]. Di Gerlando et al. (2019) performed a CNV detection study using ovine SNP50K Beadchip data on 468 Valle del Belice sheep and identified a total of 365 CNVRs [66]. Taghizadeh et al. (2022) have conducted a CNV detection study using ovine SNP50K BeadChip data on 192 genomic samples from three breeds, including 96 Baluchi sheep, 47 Lori-Bakhtiari sheep (fat-tailed), and 47 Zel sheep (thin-tailed), resulting in the identification of 515 CNVRs [67]. Ma et al. (2017) used the Illumina Ovine SNP 600 K BeadChip array for genome-wide detection of CNVs in 48 Chinese Tan sheep and identified a total of 1296 CNVRs, with 553 (42%) detected in only one animal [68]. Wang et al. (2020) performed a CNV detection study using ovine SNP600K Beadchip data on 40 Hu sheep (a highly prolific breed native to China), 165 DS sheep (a synthetic line developed from hybridizing Australian Suffolk sheep and Chinese Hu sheep), and 65 SHH sheep (a crossbreed between DS sheep and Chinese Kazakh sheep) and identified a total of 919 CNVRs [69]. Salehian-Dehkordi et al. (2021) performed a CNV detection study using ovine SNP600K Beadchip data on 2059 sheep from 67 populations worldwide and identified a total of 1217 CNVRs [70]. Many factors may result in the low frequencies of the detected CNVRs in these studies, such as reference genome completeness and variant calling algorithms. 

We evaluated our results by comparing them with previously published sheep CNVs. Since some earlier studies are based on the Ovis_aris_1.0 genome build, we first converted these results from Ovis_aris_1.0 to Ovis_v3.1 using the UCSC LIFTOVER tool [71] and then compared them with our results. In this comparison, we only considered the CNVRs on autosomes, as the CNVRs on the X chromosome were excluded in some studies. The detailed comparison results are shown in Table 2. 

The comparison results indicate that our findings are more aligned with CNVRs based on the Ovine HD SNP array compared to CNVRs identified using other platforms. When comparing our study with Zhu et al. [24] and Ma et al. [68], both of which used HD SNP for CNV detection, we found 184 CNVRs in our study overlapped with those reported by Ma, with a total length of 24.4 Mbps, and 165 CNVRs with a length of 9.31 Mbs overlapped with those reported by Zhu et al. [24]. However, when comparing the CNVR findings based on the 50K SNPs, only three CNVRs, totalling 0.45 Mbs in length, and 15 CNVRs, totalling 1.69 Mbs in length, overlapped with those reported by Ma et al. [68] and Liu et al. [72], respectively. Notably, 66 CNVRs with a total length of 17.85 Mbs, which were reported by Yang et al. [65] based on the 50K SNPs, were also identified in our study. This variation may be attributed to the larger sample size of this study, which far exceeds that of other studies. Additionally, when compared to previously reported CNVR findings using the CGH array, only 65 CNVRs with a total length of 5.82 Mbps were identified, and CNVRs with a length of 0.05 Mbps were identified in our study overlapped with Jenkins et al. [73] and Hou et al. [74]. Notably, there are no overlapping CNVRs between our study and Fontanesi et al. [75]. The inconsistency between the results of different studies illustrated that the CNVRs identified using different populations and different technology platforms could lead to different results. It also suggested there is still a vast number of CNVs in the sheep genome that have not been discovered. 

**Table 2:** Comparison of CNVRs on autosomes between this study and results from other studies of the Ovine genome.

		Findings from Different Studies							CNVRs Overlapping with This Study			
Platform	Study	Species	Sample	Count	Total Length (Mbs)	Loss	Gain	Both	Count	Percentage of Count	Total Length (Mbs)	Percentage Length
**CGH**	Jenkins et al. (2016) [73]	Texel, Coopworth, Perendale,	30	3488	67.6	2023	1325	140	65	9.90%	5.82	13.20%
		Romney, and Merino										
	Fontanesi et al. (2011) [75]	Bagnolese, Comisana, Laticauda, Massese, Sarda, and Valle del Belice	11	135	10.5	75	59	1	0	0%	0	0%
	Hou et al. (2015) [74]	Mongolian sheep, Kazakh	122	51	15.55	21	23	7	1	0.15%	0.05	
		Sheep, Tibetan sheep, Hu sheep										
**SNP50**	Yang et al. (2018) [65]	Sixty-eight breeds from Africa, America, Asia, and Europe	2254	619	197	-	-	-	66	10.06%	17.85	40.66%
	Di Gerlando et al. (2019) [66]	Valle del Belice sheep	468	365	118.36	320	43	2				
	Goyache et al. (2021) [76]	Djallonké (West African Dwarf) sheep	184	63	82.5	36	7	20				
	Taghizadeh et al. (2022) [67]	Baluchi sheep Lori-Bakhtiari sheep Zel sheep	192	515	73.85	141	364	10				
	Ma et al. (2015) [77]	Dorper, Poll Dorset, Texel, Suffolk, South American Mutton Merino, Borderdale, Gansu Alpine Merino, and Gansu Morden sheep	160	111	13.75	12	99	0	3	0.45%	0.22	0.50%
	Liu et al. (2013) [72]	German Mutton, Dorper, and Sunite	100	238	60.35	219	13	6	15	2.20%	1.69	3.80%
**SNP600**	Ma et al. (2017) [68]	Chinese Tan sheep	48	1296	121.8	1173	119	5	184	28.00%	24.4	55.60%
	Zhu et al. (2016) [24]	Chinese indigenous sheep	120	490	81.04	390	93	7	165	25.10%	9.31	21.20%
	Wang et al. (2020) [69]	Hu sheep DS sheep SHH sheep	270	919	48.17	730	102	87				
	Salehian-Dehkordi et al. (2021) [70]	sheep from 67 populations all over the world	2059	1217	245	918	197	102				
	This Study	Chinese Merino sheep	288	656	43.9	519	60	77	-	-	-	-

CGH means comparative genomic hybridization arrays, SNP50 stands for SNP50K beadchip array, SNP600 represents SNP600K beadchip array.

We utilized UCSC gene annotation (http://genome.ucsc.edu) to identify genes either fully or partially overlapping with the CNVRs, resulting in the identification of 1592 genes. Interestingly, we identified the functions of some genes associated with hair follicle development and fibre diameter trait. For example, *KRT17*, located in a CNVR detected in 11 animals, is specifically involved in determining the shape and orientation of hair [78]; *MAPK1*, found within a CNVR identified in three animals, plays a critical role in the hair cycle and maintenance of hair follicle stem cell quiescence [79]; and *EPHA5*, located in a CNVR detected in three animals, encodes a protein that belongs to the ephrin receptor subfamily of the protein tyrosine kinase family [80]. Moreover, to make further use of these annotation genes, we conducted a GO analysis and identified 31 over-represented GO terms. Similar to other GO analysis results [67,81], the functions of these genes are enriched in odorant binding activity and ATP binding activity. The KEGG pathway analysis confirmed that the most significantly enriched pathway was neuroactive ligand–receptor interaction, as reported in other studies [82].

To validate the CNVRs detected by PennCNV, we performed qPCR on 11 selected CNVRs and compared the results to a control region known to be free of CNVs. We found that eight (72.7%) of our qPCR results agreed with CNVR predictions in these regions. The high percentage could be explained by the high-density probe of the Ovine HD BeadChip and the large animal resource; additionally, the stringent CNV detection criteria employed in this study, requiring at least ten consecutive SNPs, ensured that most CNVRs predicted by PennCNV closely matched the qPCR validation results. It is important to note that three CNVRs were not validated by qPCR. This discrepancy may be due to the ambiguous boundaries of CNVs inferred from the SNP array, along with potential influences from SNPs and small indels. Future CNV research using next-generation sequencing (NGS) with larger sample sizes and complementary analytical tools will provide more precise CNV structure definitions and enhance the accuracy of CNV detection.

## 5. Conclusions

To create a comprehensive CNVR map of the ovine genome, we have conducted genome-wide CNV detection using genotyping data from 285 fine wool sheep and identified 656 CNVRs, including 628 on autosomes and 28 on the X chromosome, spanning a total of 43.9 Mbs of the sheep genome. The proportion of CNVRs varied across chromosomes, ranging from 0.45% on chromosome 26 to 3.72% on chromosome 10. For functional annotation, we conducted GO and KEGG pathway analyses. The significantly enriched GO terms were related to biological functions such as odorant binding, ATP binding, and sulfuric ester hydrolase activity. KEGG analysis confirmed that the associated genes are primarily involved in pathways like neuroactive ligand–receptor interaction, axon guidance, ECM–receptor interaction, one-carbon pool by folate, and focal adhesion (*p* < 0.05). Lastly, to validate the identified CNVRs, we performed quantitative real-time PCR experiments to assess the accuracy of copy number predictions made by PennCNV. We selected 11 potential CNVRs with various predicted statuses (gain, loss, or gain–loss) for validation, and 8 of these CNVRs (IDs 68, 156, 201, 284, 307, 352, 411, 601) were successfully confirmed. This study represents a significant advancement in mapping CNVs within the ovine genome and provides a valuable resource for future research on ovine genetic variations. Moreover, our findings offer important insights into the genomic structural variations associated with wool traits in sheep. This will support the selection of fine wool traits based on CNVRs in China’s wool industry, ultimately enhancing economic outcomes and benefiting producers.

## Figures and Tables

**Table 1:** Genomic features of copy number variations in fine wool sheep (n = 285).

Type	CNV	CNVR
Total number	5101	656
Average no. of CNVs per individual	17.9	2.3
CNV size per individual	1.4 Mb	0.2 Mb
Loss	3760	519
Gain	1214	60
Gain–loss	127	77
Total length	388.1 Mbs	43.9 Mbs
Average size	76.1 Kbs	66.9 Kbs
Median size	55.2 Kbs	51.1 Kbs

Note: CNVRs come from merging overlapping CNVs.

## Data Availability

The data supporting the findings of this study are provided within the article. Additional datasets used and/or analysed during this study are available from the corresponding author upon reasonable request.

## Supplementary Materials

The following supporting information can be downloaded at: https://www.mdpi.com/article/10.3390/ani14192897/s1, Table S1: The results of CNVs identified by PennCNV; Table S2: The results of CNVRs identified by PennCNV; Table S3: Information on primer pairs for selected CNVRs and reference genes; Table S4: Results of calculated Normalized Ratio (NR) values for each CNVR; Table S5: The Gene information overlapped by CNVRs; Table S6: Significant GO terms of the genes in this study; Table S7: Significant KEGG pathways of the genes in this study.
